# Identification of hepatic steatosis among persons with and without HIV using natural language processing

**DOI:** 10.1097/HC9.0000000000000468

**Published:** 2024-06-19

**Authors:** Jessie Torgersen, Melissa Skanderson, Farah Kidwai-Khan, Dena M. Carbonari, Janet P. Tate, Lesley S. Park, Debika Bhattacharya, Joseph K. Lim, Tamar H. Taddei, Amy C. Justice, Vincent Lo Re

**Affiliations:** 1 Department of Medicine, Perelman School of Medicine, University of Pennsylvania, Philadelphia, Pennsylvania, USA; 2 Department of Biostatistics, Epidemiology, and Informatics, Center for Clinical Epidemiology and Biostatistics, Center for Real-world Effectiveness and Safety of Therapeutics, Perelman School of Medicine, University of Pennsylvania, Philadelphia, Pennsylvania, USA; 3 Department of Medicine, Yale School of Medicine, New Haven, Connecticut, USA; 4 Department of Medicine, VA Connecticut Healthcare System, West Haven, Connecticut, USA; 5 Department of Epidemiology & Population Health, Stanford University School of Medicine, Stanford, California, USA; 6 Department of Medicine, VA Greater Los Angeles Healthcare System and David Geffen School of Medicine at UCLA, Los Angeles, California, USA; 7 Department of Epidemiology and Public Health, Division of Health Policy and Management, Yale School of Public Health, New Haven, Connecticut, USA

## Abstract

**Background:**

Steatotic liver disease (SLD) is a growing phenomenon, and our understanding of its determinants has been limited by our ability to identify it clinically. Natural language processing (NLP) can potentially identify hepatic steatosis systematically within large clinical repositories of imaging reports. We validated the performance of an NLP algorithm for the identification of SLD in clinical imaging reports and applied this tool to a large population of people with and without HIV.

**Methods:**

Patients were included in the analysis if they enrolled in the Veterans Aging Cohort Study between 2001 and 2017, had an imaging report inclusive of the liver, and had ≥2 years of observation before the imaging study. SLD was considered present when reports contained the terms “fatty,” “steatosis,” “steatotic,” or “steatohepatitis.” The performance of the SLD NLP algorithm was compared to a clinical review of 800 reports. We then applied the NLP algorithm to the first eligible imaging study and compared patient characteristics by SLD and HIV status.

**Results:**

NLP achieved 100% sensitivity and 88.5% positive predictive value for the identification of SLD. When applied to 26,706 eligible Veterans Aging Cohort Study patient imaging reports, SLD was identified in 72.2% and did not significantly differ by HIV status. SLD was associated with a higher prevalence of metabolic comorbidities, alcohol use disorder, and hepatitis B and C, but not HIV infection.

**Conclusions:**

While limited to those undergoing radiologic study, the NLP algorithm accurately identified SLD in people with and without HIV and offers a valuable tool to evaluate the determinants and consequences of hepatic steatosis.

## INTRODUCTION

Steatotic liver disease (SLD), defined by hepatic triglyceride content >5% of total liver weight, manifests as a spectrum ranging from simple hepatic steatosis to steatohepatitis with or without liver fibrosis. It often goes undetected in clinical care, but recent estimates suggest that SLD occurs in 13%–32% of the general population.^1^ SLD has emerged as the second leading cause of liver dysfunction requiring transplantation in the United States,^2,3^ and current evidence has suggested that SLD is more common among people with HIV (PWH) due to viral-mediated mechanisms or metabolic dysfunction–associated with antiretroviral therapy.^4–7^ However, prevalence estimates have varied widely, due in part to small sample sizes, use of insensitive identification methods such as International Classification of Diseases (ICD) codes, varying prevalence of coinfection with viral hepatitis, or reliance on liver biopsy for ascertainment of steatosis, limiting generalizability.^8–10^ Prospective epidemiologic studies have been hindered by the need for accurate evaluation of hepatic parenchyma. As a result, the epidemiology of SLD remains unclear, and it is unknown how this differs for PWH.

Despite the pathologic definition, noninvasive methods to identify SLD are routinely employed in clinical settings and offer a means to study its epidemiology in large, real-world cohorts.^11^ Imaging modalities, including ultrasound (US), CT, and MRI, can accurately identify the presence of liver fat with sensitivity and specificity ranging from 73%–96% and 91%–100%, respectively, compared to liver biopsy.^12–14^ Reports from these imaging studies offer a potentially invaluable resource enabling population-representative cohort studies to evaluate the frequency, determinants, and consequences of SLD. Repositories of imaging reports recorded as text fields within electronic health records (EHRs) may be analyzed retrospectively to define populations with SLD and support the identification of relevant risk factors and associated outcomes, including hepatic decompensation and HCC. However, methods to support the analyses of these text fields have yet to be validated.^15^

Natural language processing (NLP) algorithms offer potentially valuable tools to systematically identify discrete text from vast repositories of unstructured data. NLP could systematically identify SLD through algorithms developed to automatically extract relevant diagnoses, keywords, and text through pattern matching and language analyses while incorporating logic rules to ensure appropriate terminology and modifiers incorporated in structured output. NLP algorithms have previously yielded promising results in clinical radiology,^16^ but the ability of NLP algorithms to identify SLD from US, CT, or MRI reports among PWH has not been evaluated. Since PWH have differing risk profiles for SLD than people without HIV (PWoH), the accuracy of NLP may differ by HIV status. We therefore developed and examined the performance of an NLP algorithm to identify patients with SLD using reports of radiographic imaging studies inclusive of the liver. Because PWH are perceived to have a greater risk of SLD than PWoH in part due to altered immune function and antiretroviral adverse effects,^17–19^ we then applied this tool to a large population of PWH and PWoH to evaluate the association between HIV status and SLD.

## METHODS

### Study design and setting

We conducted a cross-sectional study among patients in the Veterans Aging Cohort Study (VACS), an ongoing prospective cohort of PWH and 1:2 age-, sex-, race/ethnicity-, and clinical site–matched PWoH in care within Veterans Health Administration (VA) facilities across the United States.^20^ Data available included hospital and outpatient diagnoses (recorded using ICD-9 and ICD-10 codes), procedures (recorded using Current Procedural Terminology codes), imaging reports, laboratory results, and dispensed medications. Data were queried from the national VA Corporate Data Warehouse for NLP development and creation of analytic data sets.

### Institutional review

The study was approved by the Institutional Review Boards of the VA Connecticut Healthcare System and Yale University and was conducted under a waiver of informed consent per 45 CFR §46.117(c). All research was conducted in accordance with both the Declarations of Helsinki and Istanbul.

### Study patients

PWH and PWoH were eligible if their enrollment date in VACS was between October 1, 2001, and September 30, 2017. This date range was selected to capture more of the modern antiretroviral therapy era and avoid undue influence of older antiretrovirals (eg, dideoxynucleoside analogs: stavudine, didanosine, zidovudine, and zalcitabine), which are associated with SLD.^21–23^ Patients were included in the NLP analysis if they were in VA care for at least 2 years before the completion of a US, CT, or MR study that included the liver since these imaging modalities validly identify SLD.^12–14^ We defined the index date as the date of the first eligible clinical imaging study performed on or after October 1, 2001. If more than 1 clinical imaging study was completed on the index date, the first study completed on that date was selected for inclusion. Prevalent diagnoses and laboratory results recorded within 2 years before the index date were also collected.

### Data processing and NLP algorithm development

**FIGURE 1:**
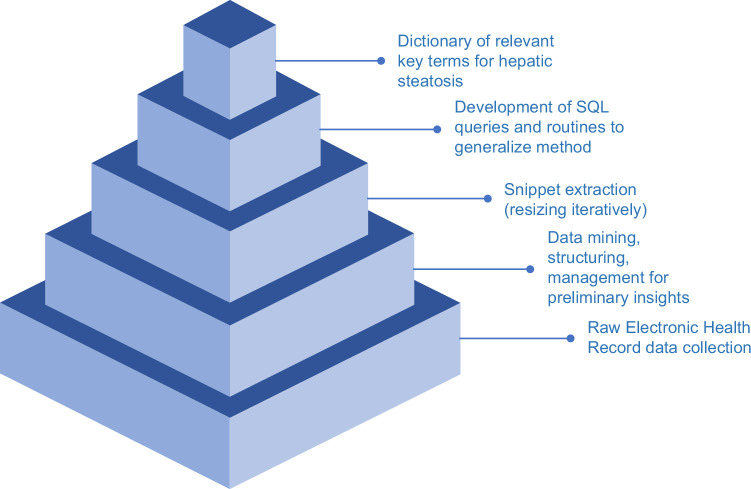
Natural language processing development for the identification of steatotic liver disease. Sequential steps depicted include preprocessing of raw data, concept search, and snippet generation to derive the dictionary comprised of the final corpus of steatotic liver disease snippets.

We applied big data management and querying techniques utilizing SQL Server analysis, .NET, and other data warehousing and management tools embedded within the EHRs at the Corporate Data Warehouse for unstructured radiology text preprocessing. Figure 1 represents a simplified view of the steps involved in the NLP algorithm development and refinement. This process focused on data collection, followed by the creation of relevant objects and algorithms, aiming to maximize generalizability and interoperability to allow for external use of the tool across multiple computing environments. All clinical imaging reports from patients were identified through query of patients’ EHRs. The extracted unstructured reports were in free-text, narrative format.

Hepatic steatosis key terms identified for extraction included “fatty” and “liver,” or “hepat”; “steatosis”; “steatotic”; and “steatohepatitis.” To extract these terms, we programmatically created reusable stored procedures and functions in SQL to parse relevant text from radiology reports to create snippets. These snippets represent sentence or phrase fragments of continuous text surrounding key terms. The coding to extract snippets from unstructured text was completed by creating specialized SQL functions. The functions were called within queries and applied in retrieving the 30 words before and after the key term. With this methodology, a total of 122,306 radiology reports were processed, and led to the extraction of 187,981 snippets. This method of snippet generation can be replicated for other conditions using the generalizable coding method.^22^ Chart reviews were completed during algorithm development to ensure the exclusion of irrelevant imaging modalities and data.

For further refinement of hepatic steatosis key term identification, we subsequently parsed radiology reports to restrict the concept search to the free-text narrative radiologist findings, body, and/or impression section of the report to prevent inclusion of reports in which key terms were only listed in the history or clinical indication text sections. We then restricted the application of the NLP algorithm to radiology reports from US, CT, or MR imaging as these modalities have previously been validated for the identification of SLD.^12–14^ Terms with “no evidence of hepatic steatosis” and “no fatty change in the liver” were excluded (Supplemental Table S2, http://links.lww.com/HC9/A932. Terms with “fatty,” “steatosis,” and “steatotic” in reference to adjacent anatomy (ie, gallbladder and pancreas) were additionally excluded. Reports noting “possible” or “suggestive of” SLD in the findings or impression sections were classified as positive findings. Final text snippets served as the dictionary of relevant key terms for SLD (Supplemental Table S1, http://links.lww.com/HC9/A932).

### Validation of NLP algorithm

To assess the accuracy of SLD identification utilizing this NLP algorithm, we performed a manual chart review on a sample of patients identified. Assuming an SLD prevalence of 30%, we calculated that 378 patients would be needed to determine at least 95% sensitivity and 80% specificity with a 95% CI of ±10%.^24^ Four hundred text reports with the key terms “fatty,” “steatosis,” “steatotic,” or “steatohepatitis” were randomly selected across the study period for the validation of the NLP algorithm. A manual review of each text report from the radiologist’s image assessment was performed by 3 liver disease experts (Tamar H. Taddei, Vincent Lo Re III, and Jessie Torgersen), who confirmed the presence of SLD (defined by the presence of hepatic steatosis, steatohepatitis, steatotic, or fatty liver) within the imaging reports. We then randomly selected and reviewed an additional 400 text reports from liver clinical imaging reports across the study period without any of the 4 hepatic steatosis key terms identified.

### Data collection

VACS includes EHR data for its patients longitudinally over a 20-year period. The data have been cleaned and curated over many years and validated through several analyses. In addition to the unstructured text notes, we collected demographic and clinical variables within 2 years prior, but closest, to the index date. These included age, sex, race/ethnicity, body mass index (BMI), HIV status, and selected comorbidities.^25^ The comorbidities were defined by 1 hospital or 2 ambulatory ICD-9/10 diagnoses and included diabetes mellitus, hypertension, chronic pulmonary disease (including asthma, bronchitis, bronchiectasis, chronic obstructive pulmonary disease, emphysema, toxin-induced or radiation-induced lung disease, and pulmonary hypertension), chronic kidney disease, alcohol use disorder, HBV infection, and HCV infection.

### Statistical analysis

We first determined the performance characteristics with 95% CIs of the NLP algorithm for the identification of SLD within imaging reports compared to manual clinician review of the reports. Sensitivity (ie, the proportion of cases identified by NLP given that the patient has SLD), positive predictive value (PPV; ie, the proportion with SLD among all cases identified by NLP), specificity (ie, the ability of NLP to correctly exclude those who do not have SLD reported), negative predictive value (NPV; ie, the proportion without SLD identified by NLP and without the condition on the radiologist report), and percent agreement (ie, the proportion of reports classified correctly by NLP among all reports included) were determined overall and by HIV status.^26^ Since semi-structured radiographic reports were increasingly utilized in later years and may impact the performance of our NLP algorithm, we additionally evaluated the performance of the NLP algorithm within early (before December 31, 2009) and late (January 1, 2010, and after) periods. These time periods were selected to reflect the period before and after a comprehensive library of templates for radiology reports was introduced by the Radiologic Society of North America.^27^ We calculated the F measure, also known as the F score, a common measurement of the predictive performance of NLP.^16^ The F measure is the harmonic mean of PPV and sensitivity, accounting for both false-positive and false-negative results, and is defined as 2 × [(PPV × sensitivity)/(PPV + sensitivity)].^28^

We then applied the NLP algorithm to PWH and PWoH who underwent liver imaging as part of clinical care. To explore whether people who underwent liver imaging were systematically different from persons who did not undergo liver imaging, we also evaluated differences in characteristics between people with and without liver imaging.

We evaluated differences in characteristics by SLD status as determined by the NLP algorithm. Since small, clinically insignificant differences may be statistically significant in analyses of large sample sizes, we evaluated the magnitude of differences in characteristics of patients by SLD and HIV status using standardized mean difference and standardized difference in proportions for continuous data and categorical variables, respectively. A standardized difference of >0.10 was considered to represent a meaningful difference between the groups.^29^

To determine if HIV infection was a significant factor associated with SLD, multivariable logistic regression was used to evaluate the association between SLD and HIV, after adjustment for age, sex, race/ethnicity, and factors traditionally associated with SLD (ie, obese BMI, diabetes, hypertension, alcohol use disorder, HBV infection, or HCV infection). The assumption of linearity of age as a continuous variable was confirmed through visual inspection of the log odds of SLD by age in the graphical display.

In a secondary analysis to explore if there might be differences in associations between traditional risk factors and SLD by HIV status, we separately developed multivariable logistic regression models to estimate odds ratios of SLD associated with risk factors of interest (ie, age, sex, race/ethnicity, BMI ≥30 kg/m^2^, diabetes, hypertension, alcohol use disorder, HCV, and HBV status) among PWH and PWoH.

Lastly, we performed a secondary analysis to evaluate the performance of NAFLD diagnostic codes (ICD-9: 571.8; ICD-10: K76.0 and K75.8)^30^ for the identification of SLD determined by the NLP algorithm. Since the diagnosis of NAFLD historically has required the exclusion of alcohol use or viral hepatitis, we restricted this analysis to patients without recorded ICD-9/-10 diagnoses of alcohol use disorder, HBV infection, and/or HCV infection. Prior work utilizing NAFLD diagnostic codes has demonstrated underutilization of the codes^31^; thus, we included any single hospital or outpatient NAFLD diagnostic code reported before or on the index date. We then used multivariable logistic regression to evaluate the association between HIV and the presence of NAFLD ICD-9/-10 diagnostic codes, after adjustment for traditional risk factors (ie, age, sex, race/ethnicity, BMI ≥30 kg/m^2^, diabetes, and hypertension) to determine if the association differed from that in the primary analysis. All statistical analyses were performed using Stata 14.1.

## RESULTS

### Performance of the NLP algorithm to identify clinician-confirmed SLD

Among the 800 sampled clinical imaging reports, the NLP algorithm identified SLD with 100% sensitivity (95% CI: 99.0%–100%), 88.5% PPV (95% CI: 85.0%–91.5%), achieving an F measure of 93.9%. The NPV and specificity of the NLP algorithm were 100% (95% CI: 99.1%–100%) and 89.7% (95% CI: 86.5%–92.3%), respectively (Table 1). The percent agreement of the NLP algorithm with clinician report review was 94.3% (95% CI: 92.4%–95.8%). Performance of the NLP algorithm did not differ by HIV status (Supplemental Table S3, http://links.lww.com/HC9/A932 and Supplemental Table S4, http://links.lww.com/HC9/A932), achieving an F measure of 92.8% and 94.2% for PWH and PWoH, respectively. In addition, the performance of the NLP algorithm did not differ across time periods (Supplemental Table S5, http://links.lww.com/HC9/A932 and Supplemental Table S6, http://links.lww.com/HC9/A932).

**TABLE 1:** Performance characteristics of hepatic steatosis key terms for confirmed steatotic liver disease within clinical imaging reports that included the liver

	Clinical expert review		
NLP algorithm	Steatotic liver disease per clinician	No steatotic liver disease per clinician	Total
Steatotic liver disease by NLP	354	46	400
No steatotic liver disease by NLP	0	400	400
Total	354	446	800
Sensitivity	100% (95% CI: 99.0%–100%)		
Specificity	89.7% (95% CI: 86.5%–92.3%)		
Positive predictive value	88.5% (95% CI: 85.0%–91.5%)		
Negative predictive value	100% (95% CI: 99.1%–100%)		
Percent agreement	94.3% (95% CI: 92.4%–95.8%)		

Abbreviation: NLP, natural language processing.

### Characteristics of PWH and PWoH by SLD status

**FIGURE 2:**
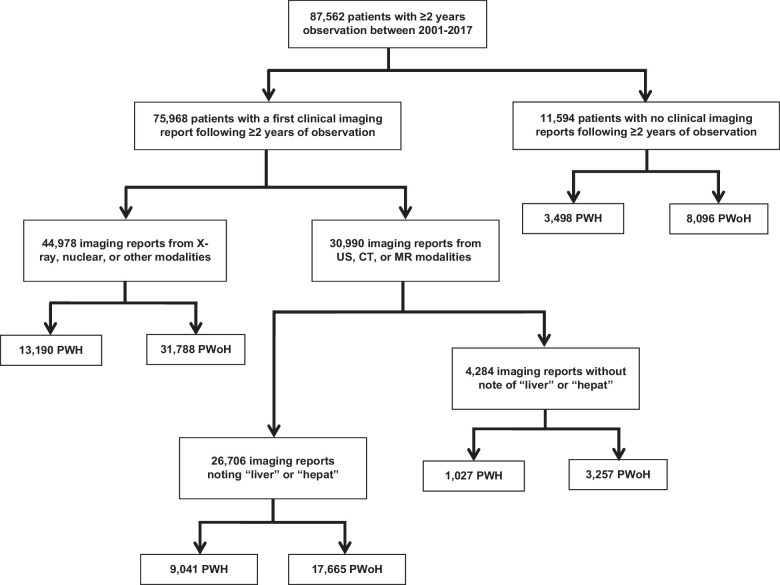
Selection of eligible patients from the Veterans Aging Cohort Study for inclusion in the study. Abbreviations: PWH, people with HIV; PWoH, people without HIV; US, ultrasound.

A total of 87,562 patients were included in the VACS between October 1, 2001, and September 30, 2017, and had at least 2 years of observation following enrollment. Of these patients, 26,706 (30.5%) underwent an US, CT, or MR study that included the liver as their first eligible imaging study (Figure 2). Of the 60,856 patients without liver imaging, 49,262 had a non-liver imaging study and 11,594 had no clinical imaging and were not included in the NLP analysis. When compared to patients with liver imaging reports, patients with no liver imaging reports were younger, Black, and had a lower prevalence of metabolic comorbidities, alcohol use disorder, and viral hepatitis (Supplemental Table S7, http://links.lww.com/HC9/A932). Among the 26,706 patients who had an US, CT, or MR study that included the liver as their first eligible imaging study, differences in characteristics by HIV status are shown in Supplemental Table S8, http://links.lww.com/HC9/A932.

SLD was identified in 6416 (71.0%) PWH and 12,879 (72.9%) PWoH (standardized difference, 0.04). Among patients with SLD, mean age, sex, and race did not differ by HIV status (Table 2). Obesity (ie, BMI ≥30 kg/m^2^), diabetes, and hypertension were more common among PWoH, while HCV and HBV infections were more common among PWH. The prevalence of alcohol use disorder did not differ by HIV status. US-based imaging was the most common modality among patients with SLD with no substantial differences in the distribution of the year of imaging study.

**TABLE 2:** Characteristics of patients with clinical imaging reports that included the liver with or without hepatic steatosis key terms identified by the natural language processing algorithm, by HIV status

	Patients with steatotic liver disease			Patients without steatotic liver disease		
Characteristic	PWH (n = 6416)	PWoH (n = 12,879)	Std diff^a^	PWH (n = 2625)	PWoH (n = 4786)	Std diff^a^
Mean (SD) age, y	49.1 (10.0)	49.8 (9.5)	0.07	49.8 (11.6)	50.8 (11.2)	0.09
Sex, male, n (%)	6241 (97.3)	12,504 (97.1)	0.01	2566 (97.7)	4637 (96.9)	0.05
Race/ethnicity, n (%)			0.04			0.09
White	2630 (41.0)	5406 (42.0)		982 (37.4)	1813 (37.8)	
Black	2889 (45.0)	5580 (43.3)		1337 (51.0)	2427 (50.7)	
Hispanic	648 (10.1)	1408 (10.9)		171 (6.5)	371 (7.8)	
Other^b^	249 (3.9)	485 (3.8)		135 (5.1)	175 (3.7)	
Body mass index ≥30 kg/m^2^, n (%)	1385 (21.6)	6135 (47.7)	0.57	381 (14.6)	1687 (35.3)	0.49
Comorbidities^c^, n (%)						
Diabetes	1315 (20.5)	4353 (33.8)	0.30	334 (12.8)	1062 (22.2)	0.25
Hypertension	3222 (50.2)	8409 (65.3)	0.31	983 (37.5)	2519 (52.6)	0.31
Pulmonary disease	955 (14.9)	2273 (17.7)	0.07	230 (8.8)	508 (10.6)	0.06
Chronic renal disease	604 (9.4)	865 (6.7)	0.10	211 (8.0)	294 (6.1)	0.07
Alcohol use disorder	1251 (19.5)	2477 (19.2)	<0.01	333 (12.7)	583 (12.2)	0.01
HBV infection	488 (7.6)	161 (1.3)	0.31	135 (5.1)	31 (0.6)	0.27
HCV infection	1947 (30.4)	2198 (17.1)	0.32	493 (18.8)	398 (8.3)	0.31
Imaging modality, n (%)			0.12			0.09
US	3646 (56.8)	6562 (51.0)		1052 (40.1)	1767 (36.9)	
CT	2435 (38.0)	5620 (43.6)		1132 (43.1)	2062 (43.1)	
MR	296 (4.6)	626 (4.9)		428 (16.3)	934 (19.5)	
Unspecified^d^	39 (0.6)	71 (0.6)		13 (0.5)	23 (0.5)	
Year of imaging study, n (%)			0.06			0.07
2001–2005	1848 (28.8)	3940 (30.6)		488 (18.6)	1001 (20.9)	
2006–2009	1953 (30.4)	3772 (29.3)		707 (26.9)	1259 (26.3)	
2010–2013	1624 (25.3)	3016 (23.4)		747 (28.5)	1247 (26.0)	
2014–2017	991 (15.5)	2151 (16.7)		683 (26.0)	1279 (26.7)	

a Standardized mean difference and standardized difference in proportions presented for continuous and categorical variables, respectively.

b Other includes Asian, American Indian, or missing categorization.

c Defined by 1 hospital or 2 ambulatory ICD-9/-10 codes.

d Report contained liver imaging results; however, we were unable to distinguish cross-sectional imaging modality as CT or MR.

Abbreviations: PWH, people with HIV; PWoH, people without HIV; Std Diff, standardized difference; US, ultrasound.

### Association between HIV infection and SLD

In multivariable logistic regression, HIV infection was not independently associated with SLD identified from clinical imaging reports (Table 3). Hispanic ethnicity, metabolic comorbidities (ie, obesity, diabetes, and hypertension), alcohol use disorder, and viral hepatitis were associated with increased odds of SLD, independent of HIV status. Increasing age and Black or other race were associated with decreased odds of SLD, independent of HIV status. Point estimates of odds ratios of SLD associated with risk factors of interest did not substantially differ by HIV status (Supplemental Table S9, http://links.lww.com/HC9/A932 and Supplemental Table S10, http://links.lww.com/HC9/A932).

**TABLE 3:** Unadjusted and adjusted odds ratios of steatotic liver disease associated with HIV infection, after adjustment for potential confounding variables, among 26,706 patients with clinical imaging reports that included the liver

Characteristic	Unadjusted odds ratio (95% CI)	Adjusted odds ratio^a^ (95% CI)
HIV	0.91 (0.86–0.96)	1.01 (0.95–1.08)
Age, per 10 y	0.92 (0.89–0.94)	0.82 (0.79–0.84)
Sex, female	1.02 (0.86–1.19)	1.09 (0.92–1.28)
Race/ethnicity		
White	Reference	Reference
Black	0.78 (0.74–0.83)	0.63 (0.59–0.67)
Hispanic	1.32 (1.19–1.46)	1.23 (1.10–1.37)
Other^b^	0.82 (0.72–0.95)	0.80 (0.69–0.92)
Body mass index ≥30 kg/m^2^	1.65 (1.55–1.74)	1.51 (1.42–1.61)
Diabetes	1.79 (1.68–1.91)	1.56 (1.45–1.67)
Hypertension	1.69 (1.61–1.79)	1.69 (1.59–1.79)
Alcohol use disorder	1.70 (1.57–1.84)	1.63 (1.50–1.77)
HBV infection	1.52 (1.28–1.81)	1.63 (1.36–1.95)
HCV infection	2.00 (1.85–2.17)	2.21 (2.03–2.40)

a Multivariable logistic regression model examining association between HIV and SLD adjusted for characteristics in the table; age, sex, and race were included in the final multivariable model because of clinical importance.

b Other includes Asian, American Indian, or missing categorization.

### Performance of NAFLD diagnostic codes for the identification of SLD by NLP

Among 18,173 patients with no diagnosis of alcohol use disorder, HBV, or HCV, 801 (4.4%) patients had a diagnosis code of NAFLD before or on the index date. NAFLD ICD-9/-10 codes had a high specificity and PPV but demonstrated poor sensitivity, NPV, and agreement compared to SLD identified by NLP (Supplemental Table S11, http://links.lww.com/HC9/A932). We found no association between HIV and NAFLD ICD-9/-10 diagnostic codes, after adjustment for age, sex, race/ethnicity, BMI ≥30 kg/m^2^, diabetes, and hypertension (Supplemental Table S12, http://links.lww.com/HC9/A932).

## DISCUSSION

We developed and validated an NLP algorithm to accurately identify SLD within reports of clinically obtained radiographic imaging studies in the VACS. The algorithm demonstrated 100% sensitivity, 88.5% PPV, and 100% NPV among a validation sample of 800 reports of US, CT, and MR-based imaging studies that included the liver. When applied to all identified patients with a liver imaging report, 19,295 (72.2%) had SLD identified, with an SLD prevalence of 71.0% among PWH and 72.9% among PWoH. After adjustment for age, sex, race/ethnicity, metabolic factors, alcohol use disorder, and viral hepatitis infection, HIV was not associated with SLD. Associations between traditional risk factors and SLD did not differ by HIV status.

Our NLP algorithm demonstrated similar or superior performance over previously published NLP algorithms, for which sensitivity ranged from 51% to 100%, PPV ranged from 89% to 96%, and F measures ranged from 64.8% to 96%.^15,32,33^ These prior studies have validated and applied NLP algorithms to VA^33,34^ and non-VA EHR data,^15,32^ similarly utilizing imaging modalities for the identification of SLD. However, our study is the first to validate and apply the algorithm to a national sample of PWH, a population with an increased risk of liver disease,^35^ and compare results to PWoH.

To our knowledge, our study of 9041 PWH and 17,665 PWoH represents the largest observational study of SLD by HIV status to date. In both groups, the prevalence of SLD identified in clinical imaging reports was more than double the estimates reported in the general population.^1^ Prior work by Natarajan et al,^34^ employing a similar methodology in a VA cohort, noted an SLD prevalence of 67.1% within a population with a comparable frequency of metabolic comorbidities. While this higher prevalence of SLD is likely due to the higher prevalence of metabolic comorbidities and viral hepatitis, factors known to increase the risk of SLD, these findings may be influenced by ascertainment bias, since analyses were limited to patients with liver imaging.^4,36–38^

We found that among people with liver imaging, HIV was not independently associated with SLD, after accounting for age, sex, race/ethnicity, metabolic comorbidities, alcohol use disorder, and viral hepatitis. Prior studies have provided conflicting results regarding the associations between HIV and hepatic steatosis and report positive,^39–42^ negative,^19,43^ and no associations,^44–47^ with differences across studies as a result of differences in diagnostic modality and population of interest. Our large, real-world observational study suggests that among people with liver imaging, HIV is not associated with SLD.

Our study had several limitations. First, while NLP offers a powerful tool to identify patients with SLD reported in clinical imaging studies, misclassification may occur by virtue of variable performance characteristics of imaging modalities as well as variable propensity for radiologists to explicitly comment on the presence or absence of hepatic steatosis key terms. Our work provides further supportive evidence that NLP offers a substantial improvement over NAFLD ICD-9/-10 codes for the identification of radiologically confirmed SLD.^31,32^ Future work including direct analysis of images is needed to identify the presence of SLD in clinical cohorts. Second, we limited our application of NLP to the first liver imaging report at least 2 years following enrollment into VACS. Nearly 70% of VACS patients did not have an eligible liver imaging study and thus were not included in the NLP analysis. Patients with liver imaging had a higher prevalence of metabolic comorbidities, alcohol use disorder, and viral hepatitis; therefore, our findings may not be generalizable to people without liver imaging. While noninvasive imaging methods, including US, CT, and MRI are widely used for the diagnosis of SLD, patients who underwent transient elastography with computed attenuation parameter assessment of liver fat were not included as an imaging modality in this analysis as it was not widely available during the study period. Third, the cross-sectional nature of our study does not capture pathologic mechanisms that may differ over time by HIV status, nor the rate at which SLD complications arise, including decompensated cirrhosis and HCC. Further work building on our NLP techniques can identify how changes in comorbidities over time may differentially impact the course of SLD by HIV status. Finally, we validated and applied an SQL-based NLP algorithm to identify steatotic liver key terms within semi-structured clinical radiology text reports. Simple, low-cost techniques like text search using SQL offer an efficient method to detect unique key terms indicative of specific conditions like hepatic steatosis. State-of-the-art NLP packages, such as MedSpaCy and sciSpaCy,^48,49^ are available to be utilized in the medical domain and operate in the spacy processing pipeline; however, such tools may offer limited improvement beyond our algorithm given the high specificity of SLD key terms. Large language models are emerging tools in clinical investigations and present an opportunity to rethink the development of artificial intelligence in medicine to capture complex domains with the integration of clinical decision support systems.^50^ While large language models offer an exciting method to analyze large radiographic repositories, the interoperability of our SQL-based NLP algorithm facilitates reproducibility within other EHRs.

## CONCLUSIONS

NLP-based tools offer the ability to accurately identify SLD in large populations of patients with and without HIV when applied to clinical imaging reports within EHRs. SLD was common within radiographic reports inclusive of the liver and was associated with a higher prevalence of comorbid diseases, including metabolic diseases and viral hepatitis. While HIV was not independently associated with SLD, further work using NLP can facilitate the evaluation of SLD risk over time as determinants and outcomes may differ by HIV status.

## Supplementary Material

SUPPLEMENTARY MATERIAL
