# Supplementary material for: Identification of hepatic steatosis among persons with and without HIV using natural language processing
Source: Hepatol Commun. 2024 Jun 19;8(7):e0468. doi: 10.1097/HC9.0000000000000468 (PMC11186806; doi:10.1097/HC9.0000000000000468)
Supplement: SUPPLEMENTARY MATERIAL [file hc9-8-e0468-s001.docx]

**Identification of Hepatic Steatosis Among Persons with and without HIV Using Natural Language Processing**

Jessie Torgersen, MD, MHS, MSCE; Melissa Skanderson, MSW; Farah Kidwai-Khan, DEng; Dena M. Carbonari, MS; Janet P. Tate, MPH, ScD; Lesley S. Park, PhD, MPH; Debika Bhattacharya, MD; Joseph K. Lim, MD; Tamar H. Taddei, MD; Amy C. Justice, MD, PhD; Vincent Lo Re III, MD, MSCE

**Table of Contents:**

Supplemental Table S1………………………………………………………………………………..2

Supplemental Table S2………………………………………………………………………………..4

Supplemental Table S3………………………………………………………………………………..5

Supplemental Table S4………………………………………………………………………………..6

Supplemental Table S5………………………………………………………………………………..7

Supplemental Table S6………………………………………………………………………………..8

Supplemental Table S7………………………………………………………………………………..9

Supplemental Table S8……………………………………………………………………………….11

Supplemental Table S9……………………………………………………………………………….13

Supplemental Table S10..…………………………………………………………………………….14

Supplemental Table S11..…………………………………………………………………………….15

Supplemental Table S12..…………………………………………………………………………….16

**Supplemental Table S1. Most common text from snippets with steatotic liver disease key terms identified utilizing natural language processing algorithm.**

| Fatty liver |
| --- |
| Fatty infiltration of the liver |
| Diffuse fatty infiltration of the liver/hepatocellular |
| hepatomegaly with fatty liver |
| liver consistent with fatty infiltration and/or hepatocellular |
| liver suggestive of fatty infiltration/hepatocellular |
| Mild fatty infiltration of the liver |
| Liver echogenic and heterogeneous, consistent with fatty infiltration/hepatocellular |
| liver as seen in fatty infiltration and/or hepatocellular |
| Diffuse fatty infiltration of the liver |
| Hepatomegaly with diffuse fatty infiltration of the liver |
| Hepatomegaly with fatty infiltration of the liver |
| liver suggesting fatty infiltration and/or diffuse hepatocellular |
| Diffuse fatty infiltration of the liver/hepatocellular |
| Hepatomegaly, with diffuse fatty infiltration of the liver |
| Hepatomegaly and fatty infiltration of the liver |
| The findings are consistent with fatty liver |
| Liver shows fatty change. No space occupying lesion in liver |
| Fatty change in liver |
| Mild fatty liver |
| Liver shows fatty change. No space-occupying lesion in liver |
| liver consistent with fatty infiltration or hepatic |
| No acute process. Fibrofatty change of the liver |
| liver likely due to fatty infiltration/hepatocellular |
| Enlarged fatty liver |
| Diffuse enlarged fatty liver |
| No change in fatty infiltration in liver |
| Mild fatty infiltration of the liver |
| Hepatomegaly with diffuse fatty infiltration versus hepatocellular |
| The findings are suggestive of fatty changes of liver |
| There is fatty infiltrate in the liver |
| Hepatomegaly, Fatty Liver |
| The findings are consistent with fatty changes of the liver |
| Fibrofatty liver |
| hepatic parenchyma consistent with cirrhosis, fatty infiltration or chronic hepatitis |
| Liver: Fatty liver |
| Diffuse fatty metamorphosis of the liver |
| There is fatty infiltration of the liver |
| hepatomegaly and fatty liver |
| The findings are consistent with fatty changes of liver |
| The findings are s\o fatty changes of liver |
| mild fatty infiltration of the liver |
| The findings are s&o fatty changes of liver |
| Probable mild fatty changes of liver |
| liver due to fatty infiltration/hepatocellular |
| The findings are consistent with mild fatty liver |
| The findings are consistent with fatty changes of liver |
| No acute process identified. Fibrofatty change of the liver |
| Diffuse fatty liver |
| The findings are consistent with fibrofatty changes of liver |
| Liver: There is fatty infiltration of the liver |
| liver due to fatty infiltration/nonspecific hepatocellular |
| liver is fatty infiltrated with no focal hepatic |
| Hepatomegaly. Fatty infiltration of the liver |
| Mild diffuse fatty infiltration of the liver |
| hepatosplenomegaly with fatty infiltration of the liver |
| Fibrofatty changes of the liver |
| Hepatomegaly and diffuse fatty infiltration of the liver |
| Fatty infiltration of liver |
| hepatomegaly with fatty infiltration |
| liver demonstrates a decrease in attenuation consistent with a fatty liver |
| hepatic fatty infiltration |
| liver consistent with fatty infiltration |
| liver which can be seen with fatty infiltration and/or hepatocellular |
| liver parenchyma consistent with fatty infiltration and/or hepatocellular |
| Hepatomegaly and diffuse fatty infiltration and/or diffuse hepatocellular |
| Hepatosplenomegaly with fatty liver |
| Hepatomegaly with diffuse fatty infiltration, unchanged |
| hepatic echogenicity consistent with fatty infiltration or hepatic |
| hepatic parenchymal disease versus fatty liver |
| liver as seen with underlying fatty infiltration and/or diffuse hepatocellular |

**Supplemental Table S2. Most common text from snippets associated with steatotic liver disease key terms negation utilizing natural language processing algorithm.**

| no evidence of hepatic steatosis |
| --- |
| no evidence of steatosis |
| no evidence of fatty liver |
| no fatty change in the liver |
| no evidence of steatosis |
| no evidence of cirrhosis or hepatic steatosis |
| no evidence of cirrhosis, steatosis |
| interval resolution of steatosis |
| no hepatic steatosis |
| there was no steatosis |
| no hepatic mass or fatty infiltration |

**Supplemental Table S3: Performance characteristics of hepatic steatosis key terms for identification of steatotic liver disease within clinical imaging reports that included the liver among people with HIV.**

| **NLP Algorithm** | **Clinical Expert Review** | |  |
| --- | --- | --- | --- |
|  | Steatotic Liver Disease  Per Clinician | No Steatotic Liver Disease Per Clinician | Total |
| Steatotic Liver Disease by NLP | 77 | 12 | 89 |
| No Steatotic Liver Disease by NLP | 0 | 166 | 166 |
| Total | 77 | 178 | 255 |
|  | | | |
| Sensitivity | 100% (95% CI:95.3-100%) | | |
| Specificity | 93.3% (95% CI:88.5-96.4%) | | |
| Positive Predictive Value | 86.5% (95% CI:77.6-92.8%) | | |
| Negative Predictive Value | 100% (95% CI: 97.8-100%) | | |
| Percent Agreement | 95.3% (95% CI:91.1-97.5%) | | |
| Abbreviations: CI, confidence interval; NLP, natural language processing. | | | |

**Supplemental Table S4: Performance characteristics of hepatic steatosis key terms for identification of steatotic liver disease within clinical imaging reports that included the liver among people without HIV.**

| **NLP Algorithm** | **Clinical Expert Review** | |  |
| --- | --- | --- | --- |
|  | Steatotic Liver Disease  Per Clinician | No Steatotic Liver Disease Per Clinician | Total |
| Steatotic Liver Disease by NLP | 277 | 34 | 311 |
| No Steatotic Liver Disease by NLP | 0 | 234 | 234 |
| Total | 277 | 268 | 545 |
|  | | | |
| Sensitivity | 100% (95% CI: 98.7-100%) | | |
| Specificity | 87.3% (95% CI: 82.7-91.1%) | | |
| Positive Predictive Value | 89.1% (95% CI:85.1-92.3%) | | |
| Negative Predictive Value | 100% (95% CI:98.4-100%) | | |
| Percent Agreement | 93.8% (95% CI:91.4-95.6%) | | |
| Abbreviations: CI, confidence interval; NLP, natural language processing. | | | |

**Supplementary Table S5: Performance of natural language processing algorithm for identification of steatotic liver disease in radiographic reports completed before December 31, 2009.**

| **NLP Algorithm** | **Clinical Expert Review** | |  |
| --- | --- | --- | --- |
|  | Steatotic Liver Disease  Per Clinician | No Steatotic Liver Disease Per Clinician | Total |
| Steatotic Liver Disease by NLP | 134 | 11 | 145 |
| No Steatotic Liver Disease by NLP | 0 | 125 | 125 |
| Total | 134 | 136 | 270 |
|  | | | |
| Sensitivity | 100% (95% CI:97.3-100%) | | |
| Specificity | 91.9% (95% CI:86.0-95.9%) | | |
| Positive Predictive Value | 92.4% (95% CI:86.8-96.2%) | | |
| Negative Predictive Value | 100% (95% CI:97.1-100%) | | |
| Percent Agreement | 95.9% (95% CI:92.8-97.9%) | | |
| Abbreviations: CI, confidence interval; NLP, natural language processing. | | | |

**Supplementary Table S6: Performance of natural language processing algorithm for identification of steatotic liver disease in radiographic reports completed after January 1, 2010.**

| **NLP Algorithm** | **Clinical Expert Review** | |  |
| --- | --- | --- | --- |
|  | Steatotic Liver Disease  Per Clinician | No Steatotic Liver Disease Per Clinician | Total |
| Steatotic Liver Disease by NLP | 220 | 35 | 255 |
| No Steatotic Liver Disease by NLP | 0 | 275 | 275 |
| Total | 220 | 310 | 530 |
|  | | | |
| Sensitivity | 100% (95% CI:98.3-100%) | | |
| Specificity | 88.7% (95% CI:84.6-92.0%) | | |
| Positive Predictive Value | 86.3% (95% CI:81.4-90.2%) | | |
| Negative Predictive Value | 100% (95% CI:98.7-100%) | | |
| Percent Agreement | 93.4% (95% CI:90.9-95.4%) | | |
| Abbreviations: CI, confidence interval; NLP, natural language processing. | | | |

**Supplemental Table S7: Characteristics of patients with liver imaging and patients without liver imaging.**

| **Characteristics** | **Liver Image**  **(n=26,706)** | **No Liver Image^a^**  **(n=60,856)** | **Std Diff^b^** |
| --- | --- | --- | --- |
| Mean (SD) age, years | 49.8 (10.2) | 48.3 (11.8) | 0.13 |
| Male sex, n (%) | 25,948 (97.2) | 58,814 (96.6) | 0.03 |
| Race, n (%)  White  Black  Hispanic  Other^c^ | 10,831 (40.6)  12,233 (45.8)  2,598 (9.7)  1,044 (3.9) | 23,606 (38.8)  29,587 (48.6)  4,348 (7.1)  3,315 (5.5) | 0.12 |
| Body mass index ≥30 kg/m^2^, n (%) | 9,588 (35.9) | 20,733 (34.1) | 0.03 |
| Comorbidities^d^, n (%) | | | |
| Diabetes | 7,064 (26.5) | 10,659 (17.5) | 0.22 |
| Hypertension | 15,133 (56.7) | 24,650 (40.5) | 0.33 |
| Pulmonary disease | 3,966 (14.9) | 5,178 (8.5) | 0.20 |
| Chronic renal disease | 1,974 (7.4) | 2,185 (3.6) | 0.17 |
| Alcohol use disorder | 4,644 (17.4) | 7,518 (12.4) | 0.14 |
| Hepatitis B virus infection | 815 (3.1) | 618 (1.0) | 0.14 |
| Hepatitis C virus infection | 5,036 (18.9) | 3,207 (5.3) | 0.43 |
| HIV | 9,041 (33.9) | 17,717 (29.1) | 0.10 |
| Abbreviations: SD, standard deviation; Std Diff, standardized difference  ^a^Includes 49,262 patients with non-liver imaging as first clinical imaging study and 11,594 with no clinical imaging. For patients with no clinical imaging, a randomly selected primary care visit served as the index date.  ^b^Standardized mean difference and standardized difference in proportions presented for continuous and categorical variables, respectively.  ^c^Other includes Asian, American Indian, or missing categorization.  ^d^Defined by one hospital or two ambulatory ICD-9/-10 diagnostic codes. | | | |

**Supplemental Table S8. Characteristics of patients with ultrasound, computed tomography, or magnetic resonance liver imaging reports, by HIV status.**

| **Characteristics** | **People with HIV**  **(n=9,041)** | **People**  **Without HIV**  **(n=17,665)** | **Std Diff^a^** |
| --- | --- | --- | --- |
| Mean (SD) age, years | 49.3 (10.5) | 50.0 (10.0) | 0.07 |
| Male sex, n (%) | 8,807 (97.4) | 17,141 (97.0) | 0.02 |
| Race, n (%)  White  Black  Hispanic  Other^b^ | 3,612 (40.0)  4,226 (46.7)  819 (9.1)  384 (4.2) | 7,219 (40.9)  8,007 (45.3)  1,779 (10.1)  660 (3.7) | 0.05 |
| Body mass index ≥30 kg/m^2^, n (%) | 1,766 (19.5) | 7,822 (44.3) | 0.55 |
| Comorbidities^c^, n (%) | | | |
| Diabetes | 1,649 (18.2) | 5,415 (30.7) | 0.29 |
| Hypertension | 4,205 (46.5) | 10,928 (61.9) | 0.31 |
| Pulmonary disease | 1,185 (13.1) | 2,781 (15.7) | 0.08 |
| Chronic renal disease | 815 (9.0) | 1,159 (6.6) | 0.09 |
| Alcohol use disorder | 1,584(17.5) | 3,060 (17.3) | 0.01 |
| Hepatitis B virus infection | 623 (6.9) | 192 (1.1) | 0.30 |
| Hepatitis C virus infection | 2,440 (27.0) | 2,596 (14.7) | 0.31 |
| Imaging modality  US  CT  MR  Unspecified^d^ | 4,698 (52.0)  3,567 (39.5)  724 (8.0)  52 (0.6) | 8,329 (47.1)  7,682 (43.5)  1,560 (8.8)  94 (0.5) | 0.10 |
| Year of imaging study  2001-2005  2006-2009  2010-2013  2014-2017 | 2,336 (25.8)  2,660 (29.4)  2,371 (26.2)  1,674 (18.5) | 4,941 (28.0)  5,031 (28.5)  4,263 (24.1)  3,430 (19.4) | 0.06 |
| Abbreviations: CT, computed tomography; MR, magnetic resonance; SD, standard deviation; Std Diff, standardized difference; US, ultrasound.  ^a^Standardized mean difference and standardized difference in proportions presented for continuous and categorical variables, respectively.  ^b^Other includes Asian, American Indian, or missing categorization.  ^c^Defined by one hospital or two ambulatory ICD-9/-10 codes.  ^d^Report contained liver imaging results, however, unable to distinguish cross-sectional imaging modality as CT or MR. | | | |

**Supplemental Table S9. Unadjusted and adjusted odds ratios of fatty liver disease (identified in clinical imaging reports including the liver) associated with traditional risk factors among people with HIV.**

| **Characteristic** | **Unadjusted Odds Ratio (95% CI)** | **Adjusted Odds Ratio^a^ (95% CI)** |
| --- | --- | --- |
| Age, per 10 years | 0.94 (0.90-0.98) | 0.83 (0.80-0.87) |
| Female sex | 1.17 (0.87-1.57) | 1.18 (0.87-1.60) |
| Race  White  Black  Hispanic  Other^b^ | Reference  0.80 (0.73-0.89)  1.41 (1.18-1.70)  0.69 (0.55-0.86) | Reference  0.62 (0.56-0.69)  1.25 (1.03-1.50)  0.66 (0.53-0.84) |
| Body mass index ≥30 kg/m^2^ | 1.61 (1.42-1.82) | 1.46 (1.28-1.66) |
| Diabetes | 1.76 (1.55-2.01) | 1.50 (1.30-1.73) |
| Hypertension | 1.68 (1.53-1.84) | 1.68 (1.52-1.87) |
| Alcohol use disorder | 1.67 (1.47-1.90) | 1.51 (1.32-1.73) |
| Hepatitis B virus infection | 1.52 (1.25-1.85) | 1.56 (1.28-1.91) |
| Hepatitis C virus infection | 1.89 (1.69-2.11) | 1.99 (1.77-2.24) |
| Abbreviations: CI, confidence interval  ^a^Multivariable logistic regression model adjusted for characteristics in the table; age, sex, and race were forced in the final multivariable model.  **^b^**Other includes Asian, American Indian, or missing categorization | | |

**Supplemental Table S10. Unadjusted and adjusted odds ratios of fatty liver disease (identified in clinical imaging reports including the liver) associated with traditional risk factors among people without HIV.**

| **Characteristic** | **Unadjusted Odds Ratio (95% CI)** | **Adjusted Odds Ratio^a^ (95% CI)** |
| --- | --- | --- |
| Age, per 10 years | 0.90 (0.87-0.93) | 0.81 (0.78-0.84) |
| Female sex | 0.93 (0.77-1.13) | 1.03 (0.84-1.26) |
| Race  White  Black  Hispanic  Other^b^ | Reference  0.77 (0.72-0.83)  1.27 (1.12-1.44)  0.93 (0.78-1.11) | Reference  0.63 (0.59-0.68)  1.23 (1.08-1.40)  0.90 (0.74-1.08) |
| Body mass index ≥30 kg/m^2^ | 1.67 (1.56-1.78) | 1.53 (1.42-1.65) |
| Diabetes | 1.79 (1.66-1.93) | 1.58 (1.45-1.72) |
| Hypertension | 1.69 (1.58-1.81) | 1.69 (1.57-1.83) |
| Alcohol use disorder | 1.72 (1.56-1.89) | 1.70 (1.53-1.88) |
| Hepatitis B virus infection | 1.94 (1.32-2.86) | 1.86 (1.24-2.79) |
| Hepatitis C virus infection | 2.27 (2.03-2.54) | 2.44 (2.17-2.74) |
| Abbreviations: CI, confidence interval  ^a^Multivariable logistic regression model adjusted for all characteristics in the table.  **^b^**Other includes Asian, American Indian, or missing categorization | | |

**Supplemental Table S11. Performance characteristics of NAFLD diagnostic codes for identification of steatotic liver disease, as defined by natural language processing algorithm, among patients with clinical imaging reports that included the liver.**

| **ICD Codes** | **NLP Algorithm** | |  |
| --- | --- | --- | --- |
|  | Steatotic Liver Disease Key Terms | No Steatotic Liver Disease Key Terms | Total |
| Presence of NAFLD ICD code | 789 | 12 | 801 |
| No presence of NAFLD ICD code | 11,644 | 5,728 | 17,372 |
| Total | 12,433 | 5,740 | 18,173 |
|  | | | |
| Sensitivity | 6.3% (95% CI: 5.9-6.8%) | | |
| Specificity | 99.8% (95% CI: 99.6-99.9%) | | |
| Positive Predictive Value | 98.5% (95% CI: 97.4-99.2%) | | |
| Negative Predictive Value | 33.0% (95% CI: 32.3-33.7%) | | |
| Percent Agreement | 35.6% (95% CI: 35.2-36.6%) | | |
| Abbreviations: CI, confidence interval; ICD, International Classification of Diseases; NAFLD, nonalcoholic fatty liver disease; NLP, natural language processing | | | |

**Supplemental Table S12. Unadjusted and adjusted odds ratios of NAFLD ICD codes associated with HIV infection, after adjustment for potential confounding variables, among 18,713 patients with clinical imaging reports that included the liver.**

| **Characteristic** | **Unadjusted Odds Ratio (95% CI)** | **Adjusted Odds Ratio^a^ (95% CI)** |
| --- | --- | --- |
| HIV | 0.69 (0.58-0.81) | 0.89 (0.74-1.06) |
| Age, per 10 years | 0.83 (0.78-0.89) | 0.75 (0.70-0.80) |
| Female sex | 1.32 (0.93-1.88) | 1.27 (0.89-1.82) |
| Race  White  Black  Hispanic  Other^b^ | Reference  0.70 (0.59-0.82)  1.21 (0.97-1.51)  1.10 (0.79-1.53) | Reference  0.63 (0.54-0.74)  1.14 (0.91-1.42)  1.08 (0.77-1.50) |
| Body mass index ≥30 kg/m^2^ | 2.23 (1.93-2.57) | 1.80 (1.54-2.11) |
| Diabetes | 1.76 (1.52-2.03) | 1.50 (1.28-1.77) |
| Hypertension | 1.50 (1.29-1.74) | 1.46 (1.23-1.73) |
| Abbreviations: CI, confidence interval; ICD, International Classification of Diseases; NAFLD, nonalcoholic fatty liver disease  ^a^Multivariable logistic regression model adjusted for characteristics in the table; age, sex, and race were forced in the final multivariable model.  **^b^**Other includes Asian, American Indian, or missing categorization | | |
